# Partially Silencing Brain Toll-Like Receptor 4 Prevents in Part Left Ventricular Remodeling with Sympathoinhibition in Rats with Myocardial Infarction-Induced Heart Failure

**DOI:** 10.1371/journal.pone.0069053

**Published:** 2013-07-09

**Authors:** Kiyohiro Ogawa, Yoshitaka Hirooka, Takuya Kishi, Tomomi Ide, Kenji Sunagawa

**Affiliations:** 1 Departments of Cardiovascular Medicine, Kyushu University Graduate School of Medical Science, Fukuoka, Japan; 2 Advanced Cardiovascular Regulation and Therapeutics, Kyushu University Graduate School of Medical Science, Fukuoka, Japan; 3 Advanced Therapeutics for Cardiovascular Diseases, Kyushu University Graduate School of Medical Science, Fukuoka, Japan; Ohio State University Medical Center, United States of America

## Abstract

**Background:**

Left ventricular (LV) remodeling and activation of sympathetic nervous system (SNS) are cardinal features of heart failure. We previously demonstrated that enhanced central sympathetic outflow is associated with brain toll-like receptor 4 (TLR4) probably mediated by brain angiotensin II type 1 receptor in mice with myocardial infarction (MI)-induced heart failure. The purpose of the present study was to examine whether silencing brain TLR4 could prevent LV remodeling with sympathoinhibition in MI-induced heart failure.

**Methodology/Principal Findings:**

MI-induced heart failure model rats were created by ligation of left coronary artery. The expression level of TLR4 in brainstem was significantly higher in MI-induced heart failure treated with intracerebroventricular (ICV) injection of hGAPDH-SiRNA than in sham. TLR4 in brainstem was significantly lower in MI-induced heart failure treated with ICV injection of TLR4-SiRNA than in that treated with ICV injection of hGAPDH-SiRNA. Lung weight, urinary norepinephrine excretion, and LV end-diastolic pressure were significantly lower and LV dimension was significantly smaller in MI-induced heart failure treated with TLR4-SiRNA than in that treated with hGAPDH-SiRNA for 2 weeks.

**Conclusions:**

Partially silencing brain TLR4 by ICV injection of TLR4-SiRNA for 2 weeks could in part prevent LV remodeling with sympathoinhibition in rats with MI-induced heart failure. Brain TLR4 has a potential to be a target of the treatment for MI-induced heart failure.

## Introduction

It has been demonstrated that activation of sympathetic nervous system (SNS) is one of the hallmarks of heart failure state, and the activation of SNS contributes to the worsening of mortality and left ventricular (LV) dysfunction [1]–[9]. In heart failure, accumulating evidence suggests that inflammatory cascade in central nervous system is one of the important pathways in the activation of SNS [10]–[12]. In the inflammatory cascade, nuclear factor-kappa B (NF-κB) is a predominant regulator for inflammatory cytokines. Thus, the signaling of NF-κB has been considered to be important in the central nervous system of heart failure [13], [14].

Inflammatory response is usually associated with the activation of innate immunity [15]. Toll-like receptor (TLR) and interleukin 1 (IL-1) receptor share a common signaling pathway leading to NF-κB activation and proinflammatory cytokines. TLRs are considered to be family of conserved pattern-recognition receptors that are linking of immune and inflammatory process [15]–[17]. After the stimulation with a ligand, the TLRs relay a signal via myeloid differentiation primary response protein 88 (MyD88) that is the common signal adaptor molecule, and trigger the downstream stimulation of NF-κB and the induction of genes that encode proinflammatory cytokines. Recently we demonstrated that TLR4 and MyD88 were increased in brainstem of myocardial infarction (MI)-induced heart failure, and that intracerebroventricular (ICV) injection of angiotensin II type 1 receptor blocker prevented LV remodeling with sympathoinhibiton and reduction of TLR4 in brainstem [10]. These results suggest that TLR4 and MyD88-mediated inflammatory responses in brainstem would be involved in the mechanisms of LV remodeling associated with brain angiotensin II type 1 receptor-evoked sympathoexcitation in MI-induced heart failure [10]. However, it has not been clarified whether direct inhibition of brain TLR4 could prevent LV remodeling with sympathoinhibition in MI-induced heart failure or not, because the no suitable direct inhibitor of TLR4 has been available.

Considering these backgrounds, in the present study, we examined whether silencing brain TLR4 by ICV injection of TLR4-SiRNA could prevent LV remodeling with sympathoinhibition in MI-induced heart failure or not.

## Results

### Effect of TLR4-SiRNA In Vitro and In Vivo

**Figure 1:**
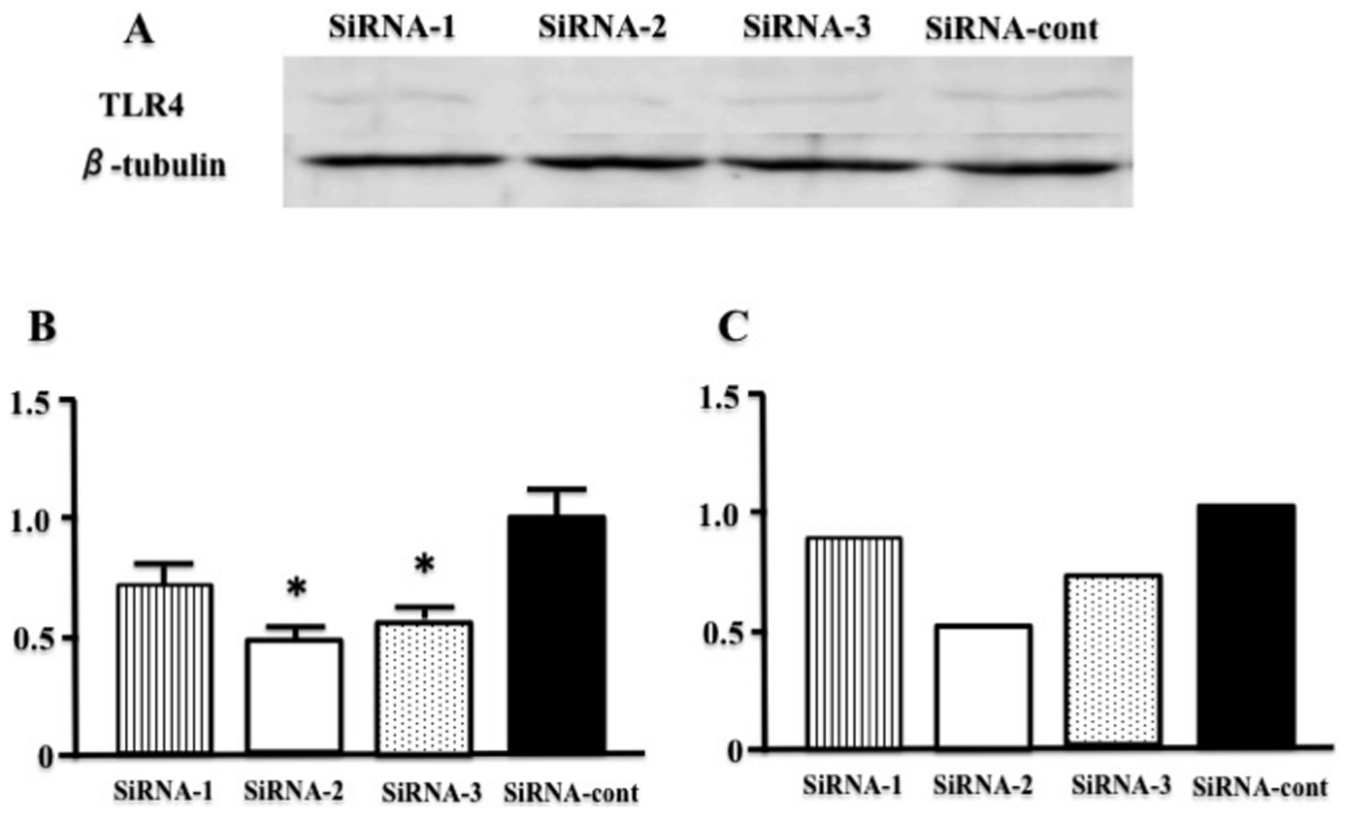
The effect of TLR4-SiRNA is determined by expression of protein and mRNA level in vitro. No 2 and 3 TLR4-SiRNA knockdown the protein expression of TLR4 compared with the sample treated with SiRNA-control (hGAPDH-SiRNA). A, B, Western blots demonstrating the expression of TLR4 in C6 cell line treated with TLR4-SiRNA 1 (SiRNA-1), 2 (SiRNA-2) and 3 (SiRNA-3), and SiRNA-control (hGAPDH-SiRNA) (P^*^ <0.01 vs SiRNA-cont, n = 3 for each). C, RT-PCR demonstrating the mRNA expression of TLR4 compared with SiRNA-cont (average, n = 2 for each).

**Figure 2:**
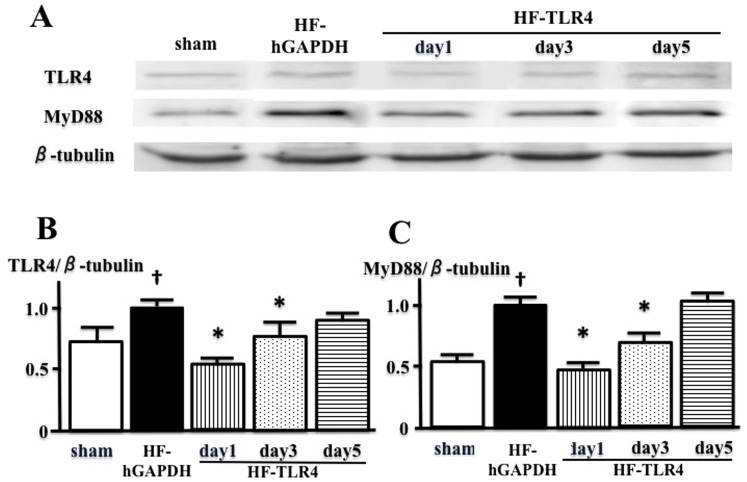
The expression of TLR4 and MyD88 in brainstem was analyzed by western blots. A, The time course of the effect of TLR4-SiRNA determined the protein expression of TLR4 and MyD88 in brainstem of sham, myocardial infarction-induced heart failure treated with intracerebroventricular (ICV) injection of hGAPDH-SiRNA (HF-hGAPDH), that treated with ICV injection of TLR4-SiRNA (HF-TLR4) at day after 1, 3, and 5. B and C, Western blots demonstrating the expression of TLR4 (B) and MyD88 (C) in brainstem. (^*^P<0.05 in HF-TLR4 vs HF-hGAPDH, n = 4 or 5 for each, ^†^P<0.05 in HF-hGAPDH vs sham, n = 5 in sham group, n = 6 in HF-hGAPDH).

**Figure 1** showed the expression of mRNA and protein of TLR4 in C6 cell line to identify the knockdown efficacy of different SiRNAs. These results suggested that SiRNA-2 was the most effective for knockdown of TLR4. Therefore, SiRNA-2 was used for further in *vivo* study. **Figure 2** showed the time course of the expression of TLR4 protein in brainstem of MI-induced heart failure treated with TLR4-SiRNA, treated with hGAPDH-SiRNA, and sham in *vivo*. The degree of the increases in the expression of TLR4 in MI-induced heart failure treated with hGAPDH-SiRNA was similar with that in MI-induced heart failure mice treated with no virus examined in our previous study [10]. Because the term of partially silencing of TLR4 in the brainstem was about 3–5 day in *vivo* (Figure **2**) and ICV injection of TLR4-SiRNA once did not change LV remodeling (data not shown), we injected TLR4-SiRNA or hGAPDH-SiRNA twice in 2 weeks (at 10 and 17 day after the coronary ligation) to determine the effects of silencing TLR4 in brainstem for 2 weeks on LV remodeling.

### Effect of ICV Injection of TLR4-SiRNA on Body Weight, Organ Weight, Infarct Size, Hemodynamics, and Activation of SNS

**Figure 3:**
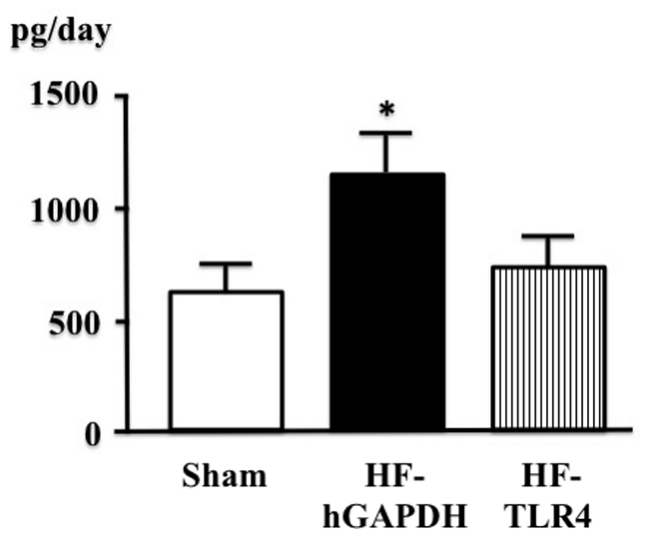
24-hour urinary norepinephrine excretion in sham, myocardial infarction-induced heart failure treated with intracerebroventricular (ICV) injection of hGAPDH-SiRNA (HF-hGAPDH), that treated with ICV injection of TLR4-SiRNA (HF-TLR4) (n = 5 for each, ^*^P<0.01 vs sham).

Table 1 shows the values for body weight (BW), organ weight, echocardiographic data, and hemodynamics. BW, lung, and heart weight were significantly lower in MI-induced heart failure treated with TLR4-SiRNA than in that treated with hGAPDH-SiRNA for 2 weeks. In histological analysis, infarct size was similar in MI-induced heart failure treated with TLR4-SiRNA and that treated with hGAPDH-SiRNA for 2 weeks. In cardiac echocardiography, LV diastolic and systolic dimension (LVDD and LVDS) was significantly smaller, and LV ejection fraction (LVEF) and cardiac output were significantly higher in MI-induced heart failure treated with TLR4-SiRNA than in that treated with hGAPDH-SiRNA for 2 weeks. LV percent fractional shortening (%FS) was not different between in MI-induced heart failure treated with TLR4-SiRNA and that treated with hGAPDH-SiRNA for 2 weeks. In hemodynamics data measured by Miller catheter, LV end-diastolic pressure (LVEDP) was significantly lower in MI-induced heart failure treated with TLR4-SiRNA than in that treated with hGAPDH-SiRNA. Maximum rate of rise of LV pressure (LV dP/dt_max_, parameter of LV systolic function) was significant higher, and highest rate of decline in LV pressure (LV -dP/dt_max_, parameter of LV relaxation and ventricular filling) was significantly lower in MI-induced heart failure treated with TLR4-SiRNA than in that treated with hGAPDH-SiRNA for 2 weeks. 24-hour urinary norepinephrine excretion was significantly lower in MI-induced heart failure treated with TLR4-SiRNA than in that treated with hGAPDH-SiRNA for 2 weeks (**Figure 3**).

**Table 1:** Physiological, echocardiographic, and hemodynamic data.

	sham	HF-hGAPDH	HF-TLR4
n	5	6	6
BW (g)	440.7±11.0	407.4±9.6*	429.0±2.5^†^
Lung/BW (mg/g)	4.2±0.1	7.7±0.4**	6.0±0.3**^†^
Heart/BW (mg/g)	4.1±0.1	5.3±0.2**	5.0±0.2**
LVDD (mm)	6.0±0.1	8.7±0.1**	7.9±0.2**^†^
LVDS (mm)	3.0±0.1	7.2±0.2**	6.1±0.1**^†^
LVEF (%) %FS (%) Cardiac output (ml/min)	87.5±1.1 51.7±0.9 52.3±5.1	43.3±1.2** 18.5±1.6** 31.3±3.8**	53.9±1.0**^†^ 21.3±1.9** 40.8±2.7**^†^
Infarct size (%)		41.1±1.3	38.5±2.1
HR (bpm)	361.5±14.5	392.9±10.1*	385.1±12.2
mBP (mmHg)	114.1±5.1	97.1±2.9*	104.7±5.0
LVEDP (mmHg)	2.4±0.3	17.9±1.1**	10.6±1.0**^†^
LV dP/dt_max_ (mmHg/ms)LV -dP/dt_max_ (mmHg/ms)	11830±684−7586±562	5983±310**−4149±612**	8335±835* ^†^−5391±774* ^†^

Data are shown as mean ± standard error of the mean.

Sham; sham operated rat, HF-hGAPDH; myocardial infarction-induced heart failure treated with hGAPDH-SiRNA, HF-TLR4; myocardial infarction-induced heart failure treate d with TLR4-SiRNA, BW; body weight, LVDD; left ventricular diastolic dimension, LVDS; left ventricular systolic dimension, LVEF; left ventricular ejection fraction, %FS; left ventricular percent fractional shortening, HR; heart rate, mBP; mean blood pressure, LVEDP; left ventricular end-diastolic pressure, LV dP/dt_max_; maximum rate of rise of left ventricular pressure, LV -dP/dt_max_; highest rate of decline in left ventricular pressure.

* P<0.01 vs sham, **P<0.05 vs sham, ^†^P<0.05 in HF-TLR4 vs HF-hGAPDH.

### Effect of ICV Injection of TLR4-SiRNA on Expression of Proinflammatory Cytokines

**Figure 4:**
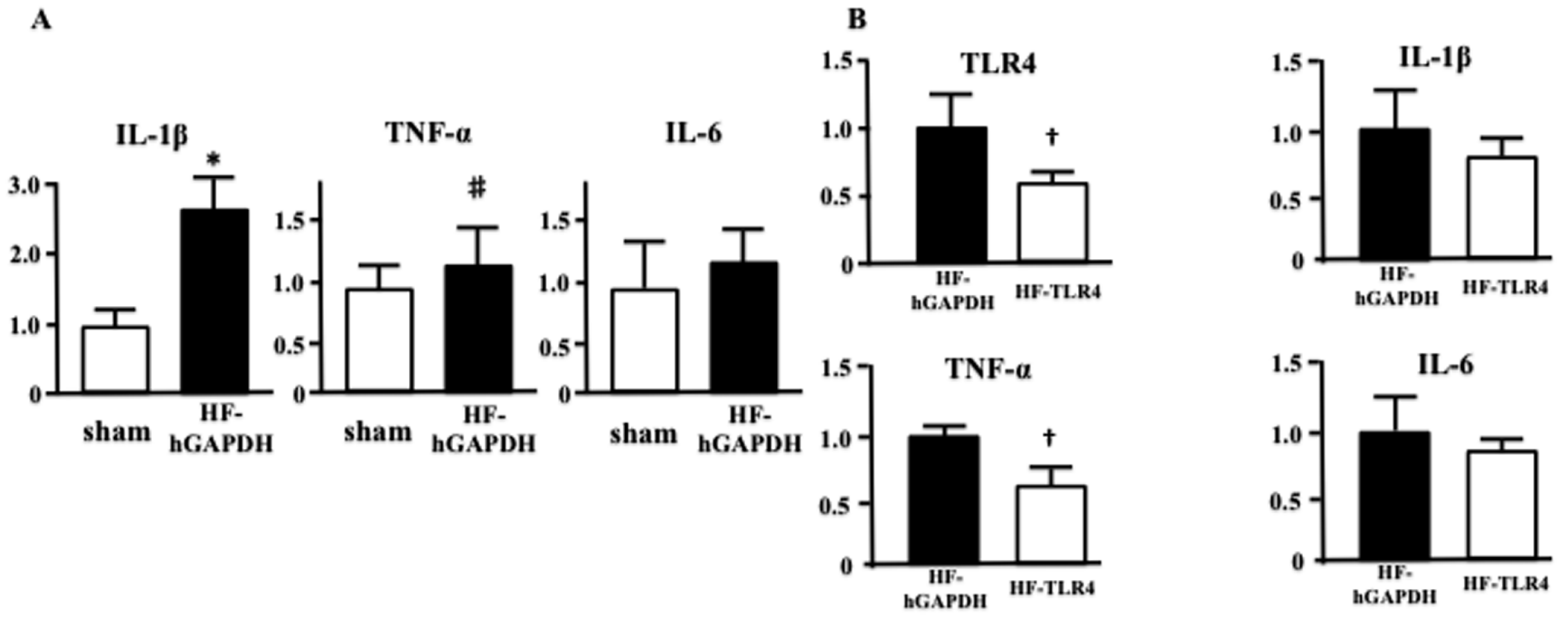
The expressions of mRNA of TLR4 and proinflammatory cytokines in brainstem were analyzed by PCR. A, The real-time reverse-transcription PCR analysis shows the mRNA expressions of the proinflammatory cytokines in brainstem of sham and myocardial infarction-induced heart failure treated with intracerebroventricular (ICV) injection of hGAPDH-SiRNA (HF-hGAPDH) (^*^P<0.01 vs sham, n = 5 for each, ^#^P<0.05 vs sham, n = 5 for each). B, The real-time reverse-transcription PCR analysis shows the mRNA expressions of TLR4 and proinflammatory cytokines in brainstem of brainstem of HF-hGAPDH and that treated with ICV injection of TLR4-SiRNA (HF-TLR4) (^+^P<0.01 vs HF-hGAPDH, n = 5 for each).

**Figure 4** showed the expressions of proinflammatory cytokines in brainstem. The expressions of IL-1β, TNF-α, and IL-6 were significantly higher in MI-induced heart failure treated with hGAPDH-SiRNA than in sham (**Figure 4A**). The expression of TLR4 in brainstem was significantly lower in MI-induced heart failure treated with TLR4-SiRNA than in that treated with hGAPDH-SiRNA for 2 weeks, as expected (**Figure 4B**). The expression of TNF-α was significantly lower in MI-induced heart failure treated with TLR4-SiRNA than in that treated with hGAPDH-SiRNA for 2 weeks (**Figure 4B**). However, the expressions of IL-1β and IL-6 were not different between in MI-induced heart failure treated with TLR4-SiRNA and that treated with hGAPDH-SiRNA for 2 weeks (**Figure 4B**).

## Discussion

The novel finding of the present study is that partially silencing brain TLR4 by ICV injection of TLR4-SiRNA for 2 weeks inhibited enhanced central sympathetic outflow and in part prevented LV remodeling in rats with MI-induced heart failure. In addition, ICV injection of TLR4-SiRNA reduced TNF-α level in brainstem. These findings suggest that brain TLR4-mediated inflammatory cascade might exacerbate in part LV remodeling with sympathoexcitation in MI-induced heart failure, and that brain TLR4 has a potential to be a target of the treatments for LV remodeling in MI-induced heart failure.

Recent several reports have suggested the possibility that the treatmenst for central inflammatory cascade could cause sympathoinhibition and improve cardiac function in heart failure [12]–[14]. Central inflammatory cascade has been considered to activate SNS [11]–[14], [18]. Furthermore, evidences have emerged the implication of the involvement of cytokines in cardiovascular diseases, such as hypertension and heart failure [19], [20]. Among the inflammatory cascade, TLRs signaling plays an important role in immune response to pathogens [15]. TLR4 is expressed in microglia and astrocytes [21]. We previously demonstrated that TLR4 expression levels and activity (Myd88 as a marker) are increased in brainstem of MI-induced heart failure, and that blockade of brain angiotensin II type 1 receptor decreased brain TLR4 with the attenuation of LV remodeling and sympathoexcitaion [10]. Although we did not investigate direct ligand for TLR4 in the previous studies, we considered that there would be a connection between brain angiotensin II type 1 receptor and brain TLR4 in mice with heart failure [10]. The results obtained in the present study are compatible with these previous reports. Moreover, because TLR4 in brainstem was partially silencing in the present study, this finding suggests that partially silencing brain TLR4 causes sympathoinhibition with the prevention of LV remodeling in MI-induced heart failure through the reduction of brain proinflammatory cytokines. Importantly, systemic infusion of TLR4-SiRNA in same dose could not produce the same results with the ICV injection of TLR4-SiRNA (data not shown). Although we did not check the effect of the ICV injection of TLR4-SiRNA on plasma and/or heart cytokines, we consider that brain TLR4-mediated inflammatory cascade might be involved in LV remodeling with sympathoexcitation in MI-induced heart failure.

Abnormal activation of SNS is associated with the prognosis of heart failure and beta-blocker therapy has significant benefits on the survival of heart failure [1]–[9]. Furthermore, activation of SNS is closely associated with LV remodeling after MI, because previous many studies have clarified that chronic beta-blocker therapy improves LV performance and reverses LV remodeling [22]–[27]. In the present study, ICV injection of TLR4-SiRNA reduced LV dimension and LVEDP, and improved LVEF and cardiac output with sympathoinhibition in MI-induced heart failure. Moreover, ICV injection of TLR4-SiRNA also improves LV dP/dt_max_ and LV −dP/dt_max_ with sympathoinhibition. These results suggest that partially silencing brain TLR4 improves LV performance such as LV contraction, relaxation, and ventricular filling via prevention of LV remodeling associated with sympathoinhibition in MI-induced heart faiure. Although there are possibilities that partially silencing TLR4 would affect plasma and heart cytokines and exacerbate smaller damages to heart independent of sympathoinhibition, we consider that partially prevention of LV remodeling in the present study would mainly obtained by sympathoinhibition directly. In a future, we would like to determine the effects of partially silencing brain TLR4 on cardiac fibrosis, because %FS is often correlated with fibrosis [28] and the release of catecholamines by the activation of SNS at the heart level is known to induce cardiac fibrosis [29]. In addition, we also should determine sarcoplasmic reticulum Ca^2+^ (SERCA2) content, because LV -dP/dt_max_ is directly linked to SERCA2 content [30].

We did ICV injection of TLR4-SiRNA twice in 2 weeks (10 and 17 day after coronary ligation), because the effective time of TLR4-SiRNA used in the present study continued for around 4 days. As shown in Figure 2, at day 1 after ICV injection of TLR4-SiRNA, the expression and activity of brain TLR4 were significantly lower in MI-induced heart failure treated with TLR4-SiRNA than in that treated with hGAPDH-SiRNA, and partially silencing brain TLR4 was continued for around 4 days. From these results, we considered that ICV injection of TLR4-SiRNA twice in 2 weeks could inhibit brain TLR4 for almost 2 weeks. However, among brain proinflammatory cytokines, IL-1β was not significantly decreased by ICV injection of TLR4-SiRNA. Actually, previous several studies suggested that brain IL-1β is increased in heart failure, and are involved in the mechanisms of sympathoexcitation [13]–[15], [21]. The discrepancy between our and previous results might be due to the other pathway of stimulating inflammatory cytokines except TLR4. The further examinations to clarify the upstream and downstream of brain TLR4 in MI-induced heart failure are necessary.

We should discuss about the mechanisms in the prevention of LV remodeling by partially silencing of brain TLR4 and implications of the results in echocardiography, because both of silencing brain TLR4 and benefits on LV remodeling were partial and superficial, and there is a discrepancy between several parameters of LV function. With regard to the mechanisms, we could not clearly demonstrate and determine the relationship between the effects of systemic and brain TLR4. We could propose just only the potential relationship between TLR4 in brainstem, sympathetic nerve activity, and LV remodeling in MI-induced heart failure. In addition, we did not check the effects of silencing brain TLR4 in sham. To examine these issues, in a future we should do chronic and brain-specific knockdown of TLR4 in sham and MI-induced heart failure, for example by Cre-Lox P system. Moreover, previously we demonstrated that ICV injection of angiotensin II type 1 receptor blocker prevent LV remodeling associated with sympathoinhibition and decreased TLR4 in brainstem of MI-induced heart failure mice [10]. Combined the previous and the present study, we could consider that brain angiotensin II type 1 receptor might exacerbate sympathoexcitation and LV remodeling through TLR4 in brainstem of MI-induced heart failure. However, it has not been clarified whether angiotensin II and/or angiotensin II type 1 receptor and TLR4 have a link in brain or not. Further studies should be done to clarify the link between angiotensin II and TLR4 in *vivo* and *vitro* study. In the aspects of cardiac echocardiography, there are discrepancies between the parameters of LV function in the present study. We consider that the discrepancies would be made because of the methodological limitations of echocardiography in rats. ICV injection of TLR4-SiRNA improves LV dP/dt and LVEDP, not infarct size and LV fractional shortening. We consider that infarct size and LV fractional shortening are varied data, and the benefits on LV dP/dt and LVEDP are meaningful to a greater extent than infarct size and LV fractional shortening. Moreover, we demonstrated that ICV injection of TLR4-SiRNA improves LVEF and cardiac output. Taking all, we consider that ICV injection of TLR4-SiRNA could improve LV performance in MI-induced heart failure.

There are several limitations in the present study. First and the most important limitation is that we could not do the really “silencing” of TLR4 in brainstem by ICV injection of TLR4-SiRNA in the present study. Although we tried to do the silencing of TLR4 by TLR4-SiRNA in higher doses, the expression of TLR4 in brainstem could not really silenced (data not shown). Because the aim of the present study was to decrease TLR4 in brainstem, we accepted ICV injection of TLR4-SiRNA. However, it is not really “silencing”. Second, we did not identify the area in the brain where the activation of TLR4 is occurred, and we also did not do the cite-specific silencing TLR4 for a longer period, especially at the nucleus involved in the cardiovascular regulation. Because of these limitations, we could not determine the benefits of silencing brain TLR4 on the survival. To clarify these issues, we should do really silencing brain TLR4 for several months by other methods in a future. Finally, we still did not find direct ligands for brain TLR4 in heart failure. Further studies are needed to clarify these important questions.

### Conclusion

The present study suggests that brain TLR4-mediated inflammatory cascade, probably not in plasma and heart, might in part exacerbate LV remodeling with sympathoexcitation in MI-induced heart failure. Although the prevention of LV remodeling and/or sympathoinhibition are necessary in the treatments for MI-induced heart failure and previous many studies have already revealed the pharmacological benefits of several agents, it is also true that we could not prevent MI-induced heart failure via LV remodeling sufficiently. The role of TLR4 in maladaptive MI-induced LV remodeling has been considered to be via inflammatory cytokine production and matrix degradation in heart [31]. Whereas now we have no available methods to inhibit or silencing brain TLR4, the present study provides the important clinical perspectives that brain TLR4 might have a potential to be a new and novel target of the treatments for MI-induced heart failure via prevention for LV remodeling additional to the usual treatments.

## Methods

### Animal

The study was reviewed and approved by the Committee on Ethics of Animal Experiments, Kyushu University Graduate School of Medical Sciences, and conducted according to the Guidelines for Animal Experiments of Kyushu University. Male Sprague-Dawley (SD) rats (250–300 g; SLC, Fukuoka, Japan) were purchased from SLC Japan (Hamamatsu, Japan).

### Cell Culture

Rat cell-lines were cultured under conventional conditions. C6 cells (RIKEN bioresource, Japan) were cultured at 37°C and 5% CO_2_, in 10% Dulbecco’s Modified Eagle Medium (DMEM) with 10% fetal bovine serum (FBS), 50 U/ml penicillin and 50 µg/ml streptomycin. For experiments, cells were plated on polystyrene cell culture dishes at a density of 3×10^6^ cells per 6-cm plate in 3 ml 10% FBS DMEM.

### SiRNA Constructs

Three different SiRNAs were made against TLR4 of rat sequence (TLR4-SiRNA), and human GAPDH sequence for the negative control (Takara Bio, Shiga, Japan). The pBAsi vector was a plasmid vector containing a RNA polymerase III (pol III) promoter for expression of SiRNA. By inserting synthesized oligo DNA sequence for hairpin RNA into the downstream of the pol III promoter, SiRNA expression vector was constructed. The sequences are as follows: 1: 5′-GACTTACAGTTTCTACGT-3′; 2: 5′-GAAGCTATAGCTTCACCAA-3′; 3: 5′-GCAGTTTCAATCGCATAGA-3′; hGAPDH: 5′-CGGGAAGCTTGTCATCAAT-3′. Plasmids were purified on columns with a Qiagen kit and quantified by A_260_ measurements. We used the plasmid vector at a concentration, 1 µg/µl *in vitro* and *vivo*.

### Transfection of TLR4-SiRNA Expression Vector

Before transfection, the cells were plated at a density of 3×10^6^ cells in 6-cm plates. Transfection with SiRNA expression vector that is specific for TLR4 or hGAPDH gene was performed.

### Induction of Myocardial Infarction-induced Heart Failure

SD rats were induced MI by ligating left coronary artery as described previously [10]. Briefly, rats were anesthetized with sodium pentobarbital (50 mg/kg intraperitoneally) under mechanical ventilation, and the thorax was opened at left intercostals space, and the left coronary artery was permanently ligated with 5–0 silk. The chest was then closed and rats were allowed to recover. Sham rats underwent the same surgical procedure without ligation. After the experimental protocols, rats were euthanized with over dose pentobarbital. The brain was removed and immediately frozen on dry ice, and the lung and heart were removed immediately and weighted. Moreover, the heart was fixed with 10% formaldehyde and cut into three parts (apex, mid and base). Each section was sliced and stained with Masson’s trichrome stain. Infarct length was measured along the endocardial and epicardial surfaces from the each LV sections. Infarct size was calculated as mean value of the infarct circumference divided by total circumference in each section (apex, mid and base) times 100. Rats with small MI size (<25%) were excluded from this study.

### Intracerebroventricular Injection

At 10 day after MI or sham operation, we divided rats into two groups, treated with ICV injection of TLR4-SiRNA or hGAPDH-SiRNA for 2 weeks. Prior at 10 day after coronary ligation, we could not do ICV infusion safely because the surgical procedures of the ICV infusion, involving anesthesia, are too hard for rats with MI-induced heart failure. In addition, we did ICV infusion again at 17 day after MI or sham operation. The rats were anesthetized with sodium pentobarbital (50 mg/kg intraperitoneally) and placed in stereotaxic instrument, and a small hole was drilled in the skull for ICV injection of TLR4-SiRNA using glass microsyringe in the right lateral ventricle (1.5 mm lateral and 1 mm posterior to the bregma, 3.8 mm in depth). 10 µg SiRNA dissolved in 10 µl water was administered for 5 minutes.

### Hemodynamics Measurements

To evaluation of LVDS, LVDD, LVEF, and %FS, cardiac echocardiography was performed at the end of the protocol under light sodium pentobarbital anesthesia (50 mg/kg intraperitoneally) with spontaneous respiration. An echocardiography system (SSD5000; Aloka, Tokyo, Japan) with a dynamically focused 7.5 MHz linear array transducer was used. LVDD amd LVDS were obtained in M-mode tracings from the short-axis view at the level of the papillary muscle. LV end-diastolic volume (LVEDV) and LV end-systolic volume (LVESV) were obtained in two-dimensional mode by taking the measurement of short-axis cross sectional areas (A) and LV length (L) (LV volume = 5/6AL, diastolic and systolic separately) [32]. LVEF was calculated by the following formula: LVEF = (LVEDV-LVESV)/LVEDV×100%. %FS was calculated as percentage in accordance with the following formula: %FS = (LVDD-LVDS)/LVDD×100(%).

To measure mean BP (mBP), LVEDP, LV dP/dt_max_, LV -dP/dt_max_, and heart rate, at the end of the protocol, rats were anesthetized with sodium pentobarbital (50 mg/kg intraperitoneally followed by 20 mg/kg per hour intravenously) under mechanical ventilation to avoid the interference of the impairment of ventilation due to lung edema and anesthesia with hemodynamic condition. A Miller catheter was inserted into the right carotid artery for measurement of mBP and heart rate, and then was advanced across the aortic valve into the LV for measurement of LVEDP, LV dP/dt_max_, and LV -dP/dt_max_.

### Evaluation of the Activity of SNS

As described previously, we measured the 24-hour urinary norepinephrine excretion as a parameter of the activity of SNS [10], [33]–[35].

### Western Blot Analysis

Western blot analysis was performed to determine the expression levels of the TLR4 (1∶250; Santa Cruz Biotechnology, CA) and MyD88 in brainstem as described previously [10], [33]–[35].

### Real-time Reverse-transcription PCR Analysis

Total RNA from brainstem was isolated using the ISOGEN (NIPPON GENE). cDNA was generated using the ReverTra Ace qPCR RT Kit (TOYOBO). Gene specific primers are as follows: TLR4: forward primer, 5′CTCACAACTTCAGTGGCTGGATTTA3′; reverse primer, 5GTCTCCACAGCCACCAGATTCTC’3′; TNF-α: forward primer, 5′ATACACTGGCCCGAGGCAAC3′; reverse primer, 5′CCACATCTCGGATCATGCTTTC3′; IL-1β: forward primer, 5′CTACCTATGTCTTGCCCGTGGAG3′; reverse primer, 5′GGGAACATCACACACTAGCAGGTC3′; IL-6: forward primer, 5′ATTGTATGAACAGCGATGATGCAC3′; reverse primer, 5′CCAGGTAGAAACGGAACTCCAGA3′; GAPDH: forward primer, 5′GGCACAGTCAAGGCTGAGAATG3′; reverse primer, 5′ATGGTGGTGAAGACGCCAGTA3′. These primers were purchased from Takara Bio (Shiga, Japan). Real-time PCR was performed using the ABI prism 7500 Sequence Detection System (Applied Biosystems, Foster City, Calif.). All data ware subsequently normalized to the glyceraldehyde-3-phosphate dehydrogenase (GAPDH) mRNA level and expressed as mRNA relative fold change.

### Statistics

Data are expressed as mean ± SEM. The statistical analyses were performed by nonpaired *t* test when comparing data between the 2 groups. A one-way analysis of variance (ANOVA) for the multiple group of ICV injection of SiRNA experiments was performed. Differences were considered to be statistically significant at a *P* value of <0.05.

## References

[1] Cohn JN, Levine TB, Olivari MT, Garberg V, Lura D, et al (1984) Plasma norepinephrine as a guide to prognosis in patients with chronic congestive heart failure. N Engl J Med 311: 819–823. 6382011 10.1056/NEJM198409273111303

[2] Ferguson DW, Berg WJ, Sanders JS (1990) Clinical and hemodynamic correlates of sympathetic nerve activity in normal humans and patients with heart failure: evidence from direct microneurographic recordings. J Am Coll Cardiol 16: 1125–1134. 2229759 10.1016/0735-1097(90)90544-y

[3] Packer M (1992) The neurohormonal hypothesis: a theory to explain the mechanism of disease progression in heart failure. J Am Coll Cardiol 20: 248–254. 1351488 10.1016/0735-1097(92)90167-l

[4] Francis GS (1989) The relationship of the sympathetic nervous system and the renin-angiotensin system in congestive heart failure. Am Heart J 118: 642–648. 2570521 10.1016/0002-8703(89)90291-3

[5] Meredith IT, Eisenhofer G, Lambert GW, Dewar EM, Jennings GL, et al (1993) Cardiac sympathetic nervous activity in congestive heart failure. Evidence for increased neuronal norepinephrine release and preserved neuronal uptake. Circulation 88: 136–145. 8391399 10.1161/01.cir.88.1.136

[6] Liu JL, Zucker IH (1999) Regulation of sympathetic nerve activity in heart failure: a role for nitric oxide and angiotensin II. Circ Res 84: 417–423. 10066676 10.1161/01.res.84.4.417

[7] Patel KP (2000) Role of paraventricular nucleus in mediating sympathetic outflow in heart failure. Heart Fail Rev 5: 73–86. 16228917 10.1023/A:1009850224802

[8] Felder RB, Francis J, Zhang ZH, Wei SG, Weiss RM, et al (2003) Heart failure and the brain: new perspectives. Am J Physiol 284: R259–276. 10.1152/ajpregu.00317.2002 12529279

[9] Kishi T, Hirooka Y (2012) Central mechanisms of abnormal sympathoexcitation in chronic heart failure. Cardiol Res Pract 2012: 847172. 22919539 10.1155/2012/847172 PMC3420224

[10] Ogawa K, Hirooka Y, Kishi T, Sunagawa K (2011) Brain AT1 receptor activates the sympathetic nervous system through toll-like receptor 4 in mice with heart failure. J Cardiovasc Pharmacol 58: 543–549. 21822148 10.1097/FJC.0b013e31822e6b40

[11] Lindley TE, Doobay MF, Sharma RV, Davisson RL (2004) Superoxide is involved in the central nervous system activation and sympathoexcitation of myocardial infarction-induced heart failure. Circ Res 94: 402–409. 14684626 10.1161/01.RES.0000112964.40701.93

[12] Yu Y, Zhang ZH, Wei SG, Serrats J, Weiss RM, et al (2010) Brain perivascular macrophages and the sympathetic response to inflammation in rats after myocardial infarction. Hypertension 55: 652–659. 20142564 10.1161/HYPERTENSIONAHA.109.142836 PMC2890291

[13] Yu Y, Zhang ZH, Wei SG, Weiss RM, Felder RB (2012) Peroxisome proliferator-activated receptor-gamma regulates inflammation and renin-angiotensin system activity in the hypothalamic paraventricular nucleus and ameliorates peripheral manifestations of heart failure. Hypertension 59: 477–484. 22083161 10.1161/HYPERTENSIONAHA.111.182345 PMC3266457

[14] Kang YM, Ma Y, Elks C, Zheng JP, Yang ZM, et al (2008) Cross-talk between cytokines and renin-angiotensin in hypothalamic paraventricular nucleus in heart failure: role of nuclear factor-kappaB. Cardiovasc Res 79: 671–678. 18469338 10.1093/cvr/cvn119 PMC2732061

[15] Akira S, Takeda K (2004) Toll-like receptor signaling. Nat Rev 4: 499–511. 10.1038/nri1391 15229469

[16] Takeda K, Kaisho T, Akira S (2003) Toll-like receptors. Annual Rev Immunol 21: 335–376. 12524386 10.1146/annurev.immunol.21.120601.141126

[17] Niizeki T, Takeishi Y, Watanabe T, Nitobe J, Miyashita T, et al (2008) Relation of serum heat shock protein 60 level to severity and prognosis in chronic heart failure secondary to ischemic or idiopathic dilated cardiomyopathy. Am J Cardiol 102: 606–610. 18721521 10.1016/j.amjcard.2008.04.030

[18] Kang YM, Zhang ZH, Xue B, Weiss RM, Felder RB (2008) Inhibition of brain proinflammatory cytokine synthesis reduces hypothalamic excitation in rats with ischemia-induced heart failure. Am J Physiol 295: H227–236. 10.1152/ajpheart.01157.2007 PMC2494768 18487441

[19] Huang BS, Leenen FH (2005) Blockade of brain mineralocorticoid receptors or Na+ channels prevents sympathetic hyperactivity and improves cardiac function in rats post-MI. Am J Physiol 288: H2491–2497. 10.1152/ajpheart.00840.2004 15615845

[20] Zhang ZH, Yu Y, Wei SG, Felder RB (2010) Centrally administered lipopolysaccharide elicits sympathetic excitation via NAD(P)H oxidase-dependent mitogen-activated protein kinase signaling. J Hypertens 28: 806–816. 20027123 10.1097/HJH.0b013e3283358b6e PMC2929929

[21] Shi P, Raizada MK, Sumners C (2010) Brain cytokines as neuromodulators in cardiovascular control. Clin Exp Pharmacol & Physiol 37: e52–57. 19566837 10.1111/j.1440-1681.2009.05234.x PMC3641564

[22] Hunt SA, Abraham WT, Chin MH, Feldman AM, Francis GS, et al (2005) ACC/AHA 2005 Guideline Update for the Diagnosis and Management of Chronic Heart Failure in the Adult: a report of the American College of Cardiology/American Heart Association Task Force on Practice Guidelines (Writing Committee to Update the 2001 Guidelines for the Evaluation and Management of Heart Failure): developed in collaboration with the American College of Chest Physicians and the International Society for Heart and Lung Transplantation: endorsed by the Heart Rhythm Society. Circulation 112: e154–e235. 16160202 10.1161/CIRCULATIONAHA.105.167586

[23] MERIT-HF Study Group (1999) Effect of metoprolol CR/XL in chronic heart failure: Metoprolol CR/XL Randomised Intervention Trial in Congestive Heart Failure (MERIT-HF). Lancet 353: 2001–2007. 10376614

[24] Dargie HJ (2001) Effect of carvedilol on outcome after myocardial infarction in patients with left-ventricular dysfunction: the CAPRICORN randomised trial. Lancet 357: 1385–1390. 11356434 10.1016/s0140-6736(00)04560-8

[25] Packer M, Fowler MB, Roecker EB, Coats AJ, Katus HA, et al (2002) Carvedilol prospective randomized cumulative survival (COPERNICUS) study group. Effect of carvedilol on the morbidity of patients with severe chronic heart failure: results of the carvedilol prospective randomized cumulative survival (COPERNICUS) study. Circulation 106: v2194–2199. 10.1161/01.cir.0000035653.72855.bf 12390947

[26] Poole-Wilson PA, Swedberg K, Cleland JG, Di Lenarda A, Hanrath P, et al (2003) Comparison of carvedilol and metoprolol on clinical outcomes in patients with chronic heart failure in the Carvedilol Or Metoprolol European Trial (COMET): randomised controlled trial. Lancet 362 7–13. 12853193 10.1016/S0140-6736(03)13800-7

[27] Willenheimer R, van Veldhuisen DJ, Silke B, Erdmann E, Follath F, et al (2005) Effect on survival and hospitalization of initiating treatment for chronic heart failure with bisoprolol followed by enalapril, as compared with the opposite sequence: results of the randomized Cardiac Insufficiency Bisoprolol Study (CIBIS) III. Circulation 112: 2426–2435. 16143696 10.1161/CIRCULATIONAHA.105.582320

[28] Zhang R, Zhang YY, Huang XR, Wu Y, Chung AC, et al (2010) C-reactive protein promotes cardiac fibrosis and inflammation in angiotensin II-induced hypertensive cardiac disease. Hypertension 55: 953–960. 20157054 10.1161/HYPERTENSIONAHA.109.140608

[29] Brouri F, Hanoun N, Mediani O, Saurini F, Hamon M, et al (2004) Blockade of beta 1- and desensitization of beta 2-adrenoreceptors reduce isoprenaline-induced cardiac fibrosis. Eur J Pharmacol 485: 227–234. 14757145 10.1016/j.ejphar.2003.11.063

[30] Trost SU, Belke DD, Bluhm WF, Meyer M, Swanson E, et al (2002) Overexpression of the sarcoplasmic reticulum Ca (2+)-ATPase improves myocardial contractility in diabetic cardiomyopathy. Diabetes 51: 1166–1171. 11916940 10.2337/diabetes.51.4.1166

[31] Timmers L, Sluijter JP, van Leulen JK, Hoefer IE, Nederhoff MG, et al (2008) Toll-like receptor 4 mediates maladaptive left ventricular remodeling and impaires cardiac function after myocardial infarction. Circ Res 102: 257–264. 18007026 10.1161/CIRCRESAHA.107.158220

[32] Gueret P, Meerbaum S, Zwehl W, Wyatt HL, Davidson RM, et al (1981) Two-dimensional echocardiographic assessment of left ventricular stroke volume: experimental correlation with thermodilution and cineangiography in normal and ischemic states. Cathet Cardiovasc Diagn 7: 247–258. 7285103 10.1002/ccd.1810070304

[33] Kishi T, Hirooka Y, Sakai K, Shigematsu H, Shimokawa H, et al (2001) Overexpression of eNOS in the RVLM causes hypotension and bradycardia via GABA release. Hypertension 38: 896–901. 11641305

[34] Kishi T, Hirooka Y, Ito K, Sakai K, Shimokawa H, et al (2002) Cardiovascular effects of overexpression of endothelial nitric oxide synthase in the rostral ventrolateral medulla in stroke-prone spontaneously hypertensive rats. Hypertension 39: 264–268. 11847195 10.1161/hy0202.102701

[35] Kimura Y, Hirooka Y, Sagara Y, Ito K, Kishi T, et al (2005) Overexpression of inducible nitric oxide synthase in rostral ventrolateral medulla causes hypertension and sympathoexcitation via an increase in oxidative stress. Circ Res 96: 252–260. 15591232 10.1161/01.RES.0000152965.75127.9d

